# Serological Analysis of IgG and IgM Antibodies against *Anaplasma* spp. in Various Animal Species of the Qinghai-Tibetan Plateau

**DOI:** 10.3390/ani12192723

**Published:** 2022-10-10

**Authors:** Jinchao Zhang, Hejia Ma, Jingkai Ai, Tongsheng Qi, Ming Kang, Jixu Li, Yali Sun

**Affiliations:** 1 State Key Laboratory of Plateau Ecology and Agriculture, Qinghai University, Xining 810016, China; 2 College of Agriculture and Animal Husbandry, Qinghai University, Xining 810016, China; 3 Qinghai Provincial Key Laboratory of Pathogen Diagnosis for Animal Diseases and Green Technical Research for Prevention and Control, Qinghai University, Xining 810016, China

**Keywords:** *Anaplasma* spp., MSP5, IgG, IgM, animals, Qinghai–Tibetan Plateau

## Abstract

**Simple Summary:**

Tick-borne pathogens are considered emergent because they cause several tick-borne diseases that threaten the health of humans and animals during tick feeding, including anaplasmosis, which is caused by *Anaplasma* spp. However, information on the carrier animals for *Anaplasma* spp. antibodies in the Qinghai–Tibetan Plateau Area is limited, and current data on the serodetection of anaplasmosis in plateau areas around the world are needed. Indirect ELISA and competitive ELISA are reliable serological tests that have been developed for the detection of *Anaplasma* infections in animals. Moreover, major surface protein 5 is a highly conserved surface protein of the *Anaplasma* genus that has proven effective as a diagnostic antigen and has been used in the serodetection of *Anaplasma* species infection in various animals with a high level of sensitivity. Hence, in this study, an rMSP5-ELISA was performed to analyze *Anaplasma* spp. IgG and IgM antibodies in potential carrier animals in the Qinghai–Tibetan Plateau Area. The results not only show the positive rate of IgG and IgM antibodies in the study animals but also indicate that there is a threat of tick biting and zoonotic pathogen infection in the vicinity of human activities in the tested areas. Our study should have major importance for identifying animals with *Anaplasma* spp. infection in the plateau area.

**Abstract:**

*Anaplasma* genus infects the blood cells of humans and animals by biting, causing zoonotic anaplasmosis. However, limited data are available on carrier animals for *Anaplasma* spp. antibodies in the Qinghai–Tibetan Plateau Area. Therefore, a serological indirect ELISA diagnostic method based on the major surface protein 5 (MSP5), derived from *Anaplasma phagocytophilum,* was developed in this study to analyze both IgG and IgM antibodies of *Anaplasma* spp. in a total of 3952 animals from the Qinghai–Tibetan Plateau, including yaks (*Bos grunniens*), cows (*Bos taurus*), cattle (*Bos taurus domesticus*), Tibetan sheep (*Ovis aries*), horses (*Equus ferus caballus*), pigs (*Sus domesticus*), chickens (*Gallus gallus domesticus*), donkeys (*Equus asinus*), stray dogs (*Canis* sp.), and stray cats (*Felis* sp.). The results showed that recombinant MSP5 protein was expressed and was successfully used to establish the indirect ELISA methods. The overall positivity for *Anaplasma* IgG and IgM antibodies was 14.6% (578/3952) and 7.9% (312/3952), respectively, and a total of 123 animals (3.1%) were both IgG- and IgM-positive. Moreover, the most prevalent *Anaplasma* IgG positivity was exhibited by donkeys (82.5%), followed by stray dogs, Tibetan sheep, pigs, chickens, horses, yaks, cows, cattle, and stray cats. The analysis for IgM antibody positivity revealed that IgM positivity was the most prevalent in the stray dogs (30.1%), followed by horses, yaks, Tibetan sheep, cows, stray cats, and cattle. Moreover, the results revealed significant differences (*p* < 0.05) at different altitudes in *Anaplasma*-specific IgG in the yaks, Tibetan sheep, and horses, and in IgM in the yaks and Tibetan sheep. In conclusion, this study is the first to demonstrate that yaks, cows, cattle, Tibetan sheep, horses, donkeys, stray dogs, stray cats, pigs, and chickens living in the Qinghai–Tibet Plateau are carrier animals for *Anaplasma* spp. IgG or IgM antibodies. The current findings provide valuable current data on the seroepidemiology of anaplasmosis in China and for plateau areas of the world.

## 1. Introduction

Tick-borne pathogens (TBPs) are considered emergent because they cause severe tick-borne diseases that threaten the health of humans and animals during tick feeding [1,2,3]. *Anaplasma* spp. are obligate intracellular bacteria from the order Rickettsiales and the family Anaplasmataceae, and are serious TBPs that cause anaplasmosis in humans and animals by infecting red and white blood cells [4,5,6,7,8]. Anaplasmosis is characterized by hemolytic anemia in various animal species and is endemic in various tropical and subtropical regions worldwide [9,10,11,12,13,14,15,16,17,18,19].

The most important *Anaplasma* species that affect animals and humans are *Anaplasma phagocytophilum*, *Anaplasma ovis*, *Anaplasma capra*, *Anaplasma marginale*, *Anaplasma bovis*, and *Anaplasma platys*. *A. phagocytophilum* is an emergent tick-borne zoonotic pathogen transmitted by *Ixodes* tick species worldwide that affects humans, dogs, cats, horses, sheep, goats, cattle, donkeys, camels, and wild boars, causing human and animal granulocytic anaplasmosis [11,12,13,14,15,16,17,18,19]. The economic impacts of *A. ovis* and the unclassified species *A. capra* are more pronounced in sheep and goats [20]. *A. marginale* is the most important cause of anaplasmosis in bovines resulting in significant economic losses, while *A. centrale* causes mild disease [21]. *A. bovis* infects monocytes in small mammals and ruminants causing anaplasmosis [22], while *A. platys* affects platelets in dogs causing infectious canine cyclic thrombocytopenia [13]. With the increase in animal and tick populations, emergent tick-borne *Anaplasma* infections are diagnosed more frequently in humans and animals [23].

Ndung’u et al. (1995) [24] and Molloy et al. (1999) [25] described competitive inhibition ELISA for the detection of anti-MSP5 antibodies, which is highly conserved and reactive to *A. marginale*, *A. centrale,* and *A. ovis*. Indirect ELISA is a reliable serological test similar to competitive ELISA that has been developed for the detection of *Anaplasma* infections in animals [26], and the key to these methods is the selection of antigens with strong specificity and high sensitivity. Major surface protein 5 (MSP5) is a highly conserved surface protein of the *Anaplasma* genus that has been proven effective as a diagnostic antigen, and this antigen has been used in the serodetection of *Anaplasma* species infection in various animals, including ruminants, equines, and canines, with a high level of sensitivity [27,28,29,30,31,32,33].

Although *Anaplasma* infections in animals have been characterized in some provinces in China [6,9,10,11,12], limited data are available on *Anaplasma* spp. antibodies in domestic and wild carrier animals in the Qinghai–Tibetan Plateau Area (QTPA). To investigate the serological epidemiology of *Anaplasma* spp. in a variety of animals that are adapted to the high altitude and cold climate of the QTPA, including yaks (*Bos grunniens*), cows (*Bos taurus*), cattle (*Bos taurus domestica*), Tibetan sheep (*Ovis aries*), horses (*Equus ferus caballus*), pigs (*Sus domesticus*), chickens (*Gallus gallus domesticus*), donkeys (*Equus asinus*), stray dogs (*Canine* sp.), and stray cats (*Felis* sp.), the serological indirect ELISA diagnostic method was developed in this study based on the MSP5 proteins derived from *Anaplasma*. This was to analyze both IgG and IgM antibodies of *Anaplasma* spp. in various animals. This assay may be of major importance in detecting *Anaplasma* spp. in animals of the plateau area.

## 2. Materials and Methods

### 2.1. Serum Collection

A total of 3952 serum samples were collected by random sampling from 10 animal species from Qinghai Province in the QTPA with geographical coordinates of 31°36′–39°19′ N and 89°35′–103°04′ E from June 2021 to February 2022. The animal species included 792 yaks, 489 cows, 451 cattle, 794 Tibetan sheep, 389 horses, 424 pigs, 220 chickens, 40 donkey, 226 stray dogs, and 127 stray cats. Information on the sampling sites is shown in Table S1. Fresh blood from different animals was collected from animals’ venous blood vessels using non-anticoagulated blood collection tubes. Subsequently, centrifugation was performed at 5000 rpm for 10 min. The serum from the supernatant was transferred to new collection tubes. Finally, all animal samples were stored in a −80 °C freezer until assayed. All procedures were carried out according to the ethical guidelines of Qinghai University.

### 2.2. Cloning and Expression of Anaplasma Major Surface Protein 5

The recombinant *Anaplasma* MSP5 protein was used to perform indirect ELISAs for detecting IgG and IgM antibodies against *Anaplasma* spp. The MSP5 gene was amplified by PCR from cDNA of Tibetan sheep blood, which was confirmed from a sample that was positive for *A. phagocytophilum* [10]. Primers that included a BamH I site (underlined) in the forward primer (FAsMSP5BamH1) 5′-CGC GGATCC TTC AGC AAA ATC GGC GAG AGG-3′ and a Not I site (underlined) in the reverse primer (RAsMSP5Not1) 5′-ATAAGAAT GCGGCCGC CTA AGA ATT AAG CAT GTG ACC-3′ for MSP5 were used [29]. The PCR products were digested with BamH I and Not I, and inserted into the pGEX-4T-1 plasmid vector, which was treated with the same restriction enzymes (Roche, Switzerland). IPTG at a final concentration of 0.2 mM was used to induce recombinant pGEX-4T-MSP5 expression in *Escherichia coli* BL21 (DE3) (New England BioLabs Inc., Ipswich, MA, USA) at 37 °C and 120 r for 12 h, and then the protein was purified with Glutathione Sepharose 4B beads (GE Healthcare Life Sciences, Pittsburgh, PA, USA) according to the manufacturer’s instructions. The final concentration of MSP5 protein was measured with a bicinchoninic acid protein assay kit (Thermo Fisher Scientific, Inc., Rockford, IL, USA) before being used.

### 2.3. Development of Indirect ELISAs for Detecting IgG and IgM Antibodies against Anaplasma spp.

Here, IgG and IgM antibodies against *Anaplasma* spp. from the study animals were detected by indirect ELISA tests based on recombinant MSP5 protein. The 0.1 μg/mL recombinant proteins were diluted in coating buffer (0.05 M carbonate–bicarbonate, pH 9.6) and incubated at 4 °C overnight to perform indirect ELISA analysis. Briefly, the next day, the ELISA plates were washed with PBS-T (0.05% Tween-20) three times, blocked with 3% skimmed milk for 1 h, then washed once. Collected sera were diluted 1:100 and incubated for 1 h at 37 °C, and the plates were washed with PBS-T six times. Then, the secondary antibodies (Table 1) were diluted 1:3000–4000 and incubated for another 1 h at 37 °C. After washing six times, the ABTS, (2,2′-azino-bis(3-ethylbenzothiazoline-6-sulfonic acid) substrate was added to the plates and incubated for 1 h at RT. The results were measured at OD 450 nm. The soluble GST protein was used as the control under consistent experimental conditions with rMSP5-ELISA. Moreover, the animal sera were confirmed as positive or negative against *Anaplasma* IgG or IgM antibodies by a commercial cELISA kit (*Anaplasma* antibody test kit, cELISA; VMRD Inc., Pullman, WA, USA) for ruminants, a commercial ELISA (*Ehrlichia equi* Antibody Kit, Helica Biosystems, Inc., Santa Ana, CA, USA) for equines, rapid in-house immunochromographic assays (SNAP^®^ 4Dx^®^ from IDEXX^®^ Laboratories, Westbrook, ME, USA) for dogs, and SPF animal sera for the remaining animal species in the study (Chunsheng, Wuhan, China) were used as controls. All positive and negative sera were kept in our laboratory at Qinghai University.

### 2.4. Data Analysis

The cut-off points were calculated by using the OD 450 values for *Anaplasma* spp. negative sera. Briefly, cut-off values = X + 3SD (X: mean values of OD 415 nm of negative controls, SD: standard deviation of OD 415 nm of negative controls). The OD 415 values of the tested animals were greater than the respective cut-off values judged as positive.

Four possible results for each type of animal in this study could be shown: IgG positivity, IgM positivity, both IgG and IgM positivity, or both IgG and IgM negativity. Therefore, the statistics in this study were presented as the after mentioned four possible results for each animal species. Moreover, this study differentiated all animals into three altitude groups: 2000–3000, 3000–4000, and 4000–5000 m altitudes to analyze the influence of the different heights above sea level on *Anaplasma* infections in the sampling areas.

To graph and analyze the data, GraphPad Prism 8 software (GraphPad Software Inc., San Diego, CA, USA) was used. The antibody prevalence and 95% confidence intervals per pathogen species were calculated using the OpenEpi program (http://www.openepi.com/Proportion/Proportion.htm) Version 3.01 accessed on 15 August 2022. The chi-squared test was used to compare proportions of positivity at different altitudes and among different animal species. The differences were considered to be statistically significant when the resulting *p*-values were lower than 0.05.

## 3. Results

### 3.1. Establishment of rMSP5-ELISA

**Figure 1:**
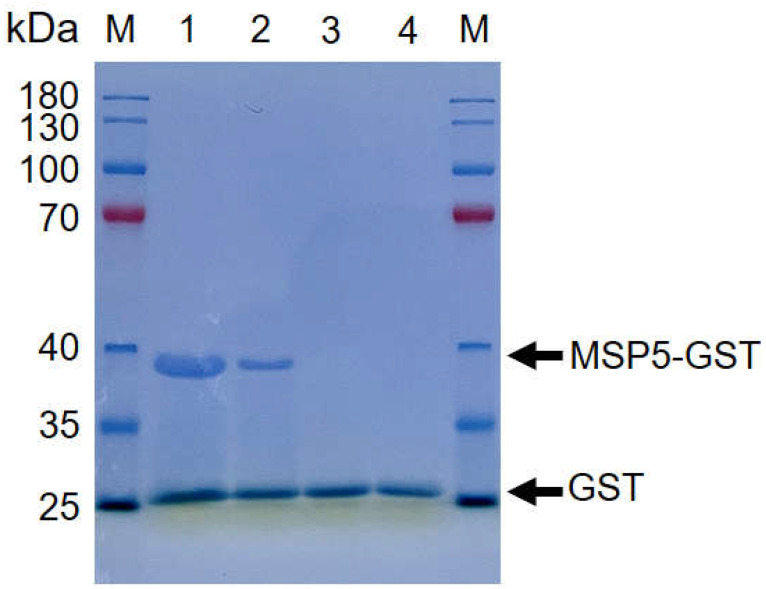
The recombinant MSP5 protein of *Anaplasma* was expressed. SDS-PAGE analysis was performed by adding 20 μL samples diluted with the loading buffer is 2 X SDS. The results showed the rMSP5-GST in fractions 1 (Protein concentration is 2 mg/μg), 2 (Protein concentration is 1 mg/μg) and the GST in fractions 3 (Protein concentration is 2 mg/μg), 4 (Protein concentration is 1 mg/μg).

**Figure 2:**
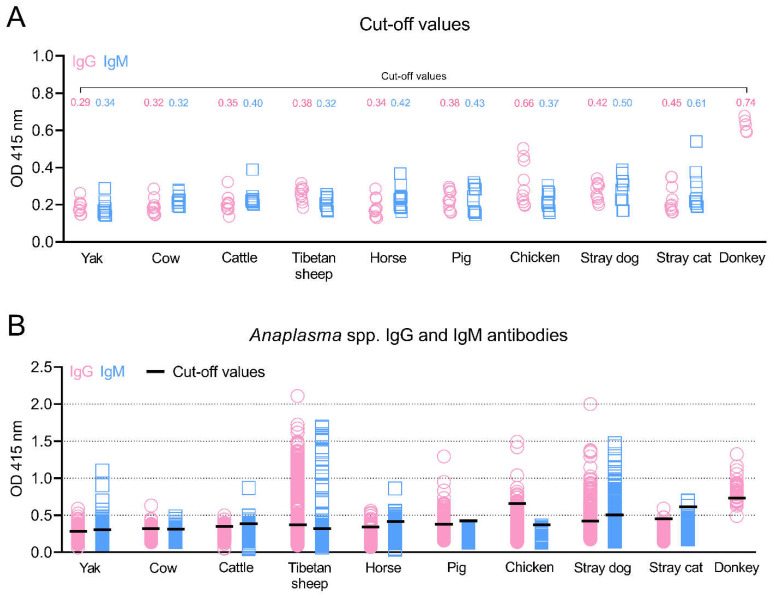
*Anaplasma* spp. IgG and IgM antibodies in various animals in the Qinghai–Tibet Plateau Area were detected by the indirect ELISA methods based on MSP5 antigens in this study. The cut-off values (**A**) and positive animals for IgG and IgM antibodies (**B**) are shown. OD: Optical Density.

In this study, the recombinant *Anaplasma* MSP5 protein was expressed (Figure 1), and used to establish indirect ELISA methods for identifying carrier animals of *Anaplasma* spp. infection in the Qinghai–Tibetan Plateau. To perform the rMSP5-based indirect ELISA in this study, the negative sera confirmed by the kits for *Anaplasma* antibody tests and the SPF animal sera were used to determine OD 415 values and to calculate cut-off values for IgG and IgM antibodies in the yaks, cows, cattle, Tibetan sheep, horses, pigs, chickens, donkeys, stray dogs, and stray cats (Figure 2A).

### 3.2. Positivity for Anaplasma IgG and IgM Antibodies

As shown in Figure 2B and Table 2, the overall positivity for *Anaplasma* IgG and IgM antibodies was 14.6% (578/3952) and 7.9% (312/3952), respectively, and a total of 123 animals (3.1%) were both IgG- and IgM-positive. A total of 10.5% (83/792) of yaks, 6.7% (33/489) of cows, 4.4% (20/451) of cattle, 29.8% (237/794) of Tibetan sheep, 11.8% (46/389) of horses, 23.8% (101/424) of pigs, 8.6% (19/220) of chickens, 82.5% (33/40) of donkeys, 49.1% (111/226) of stray dogs, and 4.7% (6/127) of stray cats were positive for at least one indicator (IgG or IgM). Moreover, the donkey was the animal with the highest IgG positivity prevalence (82.5%), followed by stray dogs, Tibetan sheep, pigs, chickens, horses, yaks, cows, cattle, and stray cats. IgM antibody positivity was most prevalent in stray dogs (30.1%), followed by horses, yaks, Tibetan sheep, cows, stray cats, and cattle. No pigs or chickens were positive for IgM antibodies against *Anaplasma*.

### 3.3. Influence of Altitude on the Positivity of Anaplasma IgG and IgM Antibodies

The results from the analysis of the influence of altitude revealed significant differences (*p* < 0.05) in *Anaplasma*-specific IgG prevalence at different altitudes in the yaks, Tibetan sheep, and horses, while IgM prevalence was significantly different in the yaks and Tibetan sheep at different altitudes (Table 3). The results revealed significant differences (*p* < 0.05) at different altitudes in *Anaplasma*-specific IgG prevalence in the yaks, Tibetan sheep, and horses and in IgM prevalence in the yaks and Tibetan sheep. There were no differences at different altitudes in the other animal species.

## 4. Discussion

The Qinghai–Tibetan Plateau Area (QTPA) is the largest plateau that has the highest average altitude on the planet, and is located in Northwestern China [10]. It has a unique and vigorous natural ecosystem due to its high altitude (with an average elevation of more than 2000 m above sea level) and cold climate (an average annual temperature below 10 °C) [11]. In specific areas, there are specialized tick species that can transmit unique pathogens. However, there are few studies on the detection of antibodies against tick-borne diseases in the Qinghai–Tibet Plateau, which is an area with much livestock production. *Anaplasma* species are zoonotic pathogens with ticks as vectors and with mammalian reservoir hosts [34]. Their transmission is closely related to the activity of ticks, and a variety of specialized species of *Ixodes* are distributed across the Qinghai–Tibet Plateau, such as *Haemaphysalis qinghaiensis*, *Dermacentor nuttalli*, and *Dermacentor silvarum* [11,12]. Importantly, the zoonotic pathogen has been detected and characterized in these ixodid ticks [35]. The *Anaplasma* genus infects the blood cells of humans and animals through biting, causing zoonotic diseases, while grazing and straying increase animal exposure to ticks. In this study, an rMSP5-ELISA was performed to analyze *Anaplasma* spp. IgG and IgM antibodies in potential carrier animals that live in proximity to humans in the QTPA. This study provides valuable current data on the epidemiology of anaplasmosis in China and in the plateau areas in the world.

MSP5 is a transmembrane protein of 210 amino acid residues that is present in all recognized *Anaplasma* species and that is highly conserved among different strains of *A. marginale*, *A. centrale*, *A. ovis* and *A. phagocytophilum* [19]. It has been used in the sero determination of *Anaplasma* infection in animals, and among the methods of serodetection, indirect ELISA and competitive ELISA are considered reliable [27,28,29,30,31,32,33,36]. The MSP5 protein in this study was expressed with 182 amino acids whose signal peptide was removed to develop an indirect ELISA for detecting antibodies against *A. marginale*, *A. centrale*, *A. ovis,* and *A. phagocytophilum* and to screen carrier animals for infection with *Anaplasma* species. Furthermore, some studies have shown that the antibodies against *A. marginale* MSP5 are recognized in both acute stages of infection and chronically infected carrier cattle [27,29]. In the current study, rMSP5-ELISA was used to detect IgG and IgM antibodies and revealed that the overall positivity for these antibodies was 14.6% and 7.9%, respectively, suggesting that tested animal species were in the acute stages of infection or are carriers of antibodies for anaplasmosis. Of course, the presence of these antibodies is inseparable from the participation of the vector ticks.

Bovine anaplasmosis, primarily caused by *A. marginale*, which can primarily cause acute anaplasmosis in adult bovines, is considered one of the most serious tick-borne diseases in ruminants [30,31]. *A. centrale*, a species closely related to *A. marginale*, is used to prevent acute anaplasmosis in several countries worldwide [30]. This study tested a total of 1732 bovines and found that 5.0% (87/1732) were *Anaplasma* IgG positive, 4.2% (73/1732) were *Anaplasma* IgM positive and 1.4% (24/1732) were both IgG and IgM positive, indicating that bovines are among those animals that can develop acute infections and middle and late infections in this plateau area. Moreover, sheep and goats can become infected with obligate intracellular bacteria of the genera *Anaplasma*, *A. phagocytophilum* and *A. ovis*, leading to the development of ovine and caprine anaplasmosis [18,28,32]. The present results showed not only a high IgG-positive rate (25.8%) but also a 5.8% IgM antibody positivity in Tibetan sheep. These results are consistent with our previous studies showing that pathogens, including *A. ovis*, *A. bovis*, *A. capra,* and *A. phagocytophilum*, were molecularly detected in yak and Tibetan sheep blood DNA samples at current sampling sites [10,11].

Tick-borne diseases in horses and donkeys mainly include equine piroplasmosis caused by *Babesia caballi* and *Theileria equi*, Lyme borreliosis caused by *Borrelia burgdorferi*, and equine granulocytic anaplasmosis caused by *A. phagocytophilum*, which are widely reported in countries across the world [7,15,16]. However, limited research has been found on the analysis of equine granulocytic anaplasmosis in the equines of China [15,16]. Horses and donkeys from areas in which *A. phagocytophilum* is endemic have a high seroprevalence of antibodies against *A. phagocytophilum* [7]. However, the difference is that the current study found a high *Anaplasma* spp. IgG positive rate (82.5%) only in donkeys, with low IgG positivity (6.9%) in horses, while IgM antibodies representing acute infection were detected in horses (6.9%) in this study. To analyze these findings, two points need to be considered: (a) equine granulocytic ehrlichiosis is a seasonal vector-borne disease closely associated with the activity of ticks from mid-spring to the end of summer, consistent with current sampling time points, and antibody positivity corresponds to the prevailing distribution of ticks of *Ixodes* genus; (b) *A. phagocytophilum* was detected in *Ixodes* ticks throughout China [37,38], including in the Qinghai–Tibet Plateau [35].

Furthermore, canine anaplasmosis caused by *A. phagocytophium* and *A. platys* is a vector-borne disease transmitted mainly via ticks, and has been extensively studied worldwide, including in China, by using molecular biology detection methods and through analysis of serological antibodies [13,39,40,41]. This study detected both *Anaplasma* spp. IgG and IgM antibodies in stray dogs and found that out of 226 dogs, 103 or 68 were positive for IgG or IgM antibodies, respectively, and 60 dogs were positive for both IgG and IgM antibodies. The results were significantly higher than the 10.1% average IgG positivity for dogs across ten provinces of China [40], which may be due to the dog samples in the current study being from stray dogs that are more likely to exposed to ticks. Moreover, cases of *A. phagocytophilum* infection in cats have been detected by molecular assays and reported in many countries [14]. The current results revealed that 3 of 127 cats were positive for *Anaplasma* IgG or IgM antibodies. This is the first report of *Anaplasma* infection in cats in China. Cats have been found to show a lower number of *A. phagocytophilum* infections in comparison with dogs [14], consistent with our results. In addition, our study investigated *Anaplasma* antibody levels in serum samples from pigs and chickens, which are two animal types for which there are few reports of *Anaplasma* infection or even detection of antibody levels. Although molecular tests in past studies may have identified unrecognized infections in wild boars and birds [17,42], our results detected only IgG antibodies and no IgM positivity in pigs and chickens of the QTPA.

The results of the current study revealed the higher *Anaplasma* IgG and IgM positive rates for yaks and higher IgG positive rates for Tibetan sheep at 3000–4000 m altitudes than at 2000–3000 m altitudes, while there were higher IgM positive rates for Tibetan sheep and higher IgG and IgM positive rates for horses at 2000–3000 m altitudes than at 3000–4000 m. This may be consistent with our unpublished investigations, which have shown that altitude did not significantly affect the distribution of ticks in the altitude region of 2000–5000 m in the Qinghai–Tibet Plateau, China. However, the distribution of ticks was related to season and humidity. The distribution of ticks significantly affects the prevalence of tick-borne pathogens in humans and animals.

## 5. Conclusions

The results of this study showed the positive rates of IgG and IgM antibodies in the study animals. Our study first demonstrated that yaks, cows, cattle, Tibetan sheep, horses, donkeys, stray dogs, stray cats, pigs, and chickens living at different altitudes on the Qinghai–Tibet Plateau were animals susceptible to *Anaplasma* spp. infection, indicating that there is a threat of tick bites and zoonotic pathogen infection in the vicinity of human and animal activities in the tested areas. These findings suggest the importance and urgency of preventing tick bites. However, the investigated IgG and IgM antibodies may vary according to several factors, such as the time of exposure, the pathogens or strains of *Anaplasma* spp. involved, and the infection status of the animals. Future studies on the interactions among vectors, animals, and pathogens utilizing molecular and serological analyses are recommended to fully elucidate the dynamics of *Anaplasma* spp. and other tick-borne pathogens in this plateau area.

## Figures and Tables

**Table 1:** Secondary antibodies used in this study.

Antibodies	Catalog, Company, Country
Rabbit Anti-Bovine IgM/HRP	bs-0327R-HRP, Bioss, China
Rabbit Anti-Bovine IgG H&L/HRP	bs-0326R-HRP, Bioss, China
Goat Anti-Cow IgG H&L/HRP	ab102154, abcam, UK
Sheep Anti-Cow IgM H&L/HRP	ab112752, abcam, UK
Rabbit Anti-Sheep IgM/HRP	ab112763, abcam, UK
Rabbit Anti-Sheep IgG H&L/HRP	AS023, Abclonal, China
Goat Anti-Horse IgM H&L/HRP	ab112879, abcam, UK
Rabbit Anti-Horse IgG/HRP	bs-0308R-HRP, Bioss, China
Rabbit Anti-Pig IgG/HRP	bs-0309R-HRP, Bioss, China
HRP Mab Pig IgM	Primadiagnostic, China
Goat Anti-Chicken IgG/HRP	bs-0310G-HRP, Bioss, China
Rabbit Anti-Chicken IgM/HRP	bs-0314R-HRP, Bioss, China
Goat Anti-Donkey IgG H&L/HRP	ab6988, abcam, UK
Goat Anti-Dog IgG H&L/HRP	ab112852, abcam, UK
Goat Anti-Dog IgM H&L/HRP	ab112835, abcam, UK
Goat Anti-Cat IgG H&L/HRP	ab112801, abcam, UK
Goat Anti-Cat IgM H&L/HRP	ab112792, abcam, UK

**Table 2:** Seropositive rates of *Anaplasma* IgG and IgM antibodies in animals in the QTPA.

Animal	No.	Total IgG-Positive (%, 95% CI)	Total IgM-Positive (%, 95% CI)	Both IgG and IgM-Positive (%, 95% CI)	Single-IgG-Positive (%, 95% CI)	Single-IgM-Positive (%, 95% CI)
Yak	792	52 (6.6, 4.8–8.3)	51 (6.4, 4.7–8.1)	20 (2.5, 1.4–3.6)	32 (4.0, 2.7–5.4)	31 (3.9, 2.6–5.3)
Cow	489	23 (4.7, 2.8–6.6)	13 (2.7, 1.2–4.1)	3 (0.6, 0.1–1.3)	20 (4.1, 2.3–5.8)	10 (2.0, 0.8–3.3)
Cattle	451	12 (2.7, 1.2–4.1)	9 (2.0, 0.7–3.3)	1 (0.2, 0.2–0.7)	11 (2.4, 1.0–3.9)	8 (1.8, 0.6–3.0)
Tibetan sheep	794	205 (25.8, 22.8–28.9)	46 (5.8, 4.2–7.4)	14 (1.8, 0.8–2.7)	191 (24.1, 21.1–27.0)	32 (4.0, 2.7–5.4)
Horse	389	27 (6.9, 4.4–9.5)	27 (6.9, 4.4–9.5)	8 (2.1, 0.6–3.5)	19 (4.9, 2.7–7.0)	19 (4.9, 2.7–7.0)
Pig	424	101 (23.8, 19.8–27.9)	0	0	101 (23.8, 19.8–27.9)	0
Chicken	220	19 (8.6, 4.9–12.3)	0	0	19 (8.6, 4.9–12.3)	0
Stray dog	226	103 (45.6, 29.1–52.1)	68 (30.1, 24.1–36.1)	60 (26.5, 20.8–32.3)	43 (19.0, 13.9–24.1)	8 (3.5, 1.1–5.9)
Stray cat	127	3 (2.4, 0.3–5.0)	3 (2.4, 0.3–5.0)	0	3 (2.4, 0.3–5.0)	3 (2.4, 0.3–5.0)
Donkey	40	33 (82.5, 70.7–94.3)	-	-	33 (82.5, 70.7–94.3)	-
Total	3952	578 (14.6, 13.5–15.7)	312 (7.9, 7.1–8.7)	123 (3.1, 2.6–3.7)	455 (11.5, 10.5–12.5)	189 (4.8, 4.1–5.4)

No.: No. of animals tested in this study; %: Prevalence; 95% CI: 95% Confidence Interval.

**Table 3:** Analysis of influence of altitude on positivity of *Anaplasma* IgG and IgM antibodies.

Antibody	Animal	2000–3000 m		3000–4000 m		4000–5000 m		*p*-Value
		Tested	Positive (%, 95% CI)	Tested	Positive (%, 95% CI)	Tested	Positive (%, 95% CI)	
IgG	Yak	319	7 (2.2, 0.6–3.8)	257	37 (14.4, 10.1–18.7)	216	8 (3.7, 1.2–6.2)	<0.0001
	Cow	489	23 (4.7, 2.8–6.6)	0	-	0	-	-
	Cattle	401	10 (2.5, 1.0–4.0)	0	-	50	2 (4.0, 1.4–9.4)	0.5457
	Tibetan sheep	147	24 (16.3, 10.4–22.3)	647	181 (28.0, 24.5–31.4)	0	-	0.0211
	Horse	40	9 (22.5, 9.6–35.4)	289	18 (6.2, 3.4–9.0)	60	0	0.0021
	Pig	424	101 (23.8, 19.8–27.9)	0	-	0	-	-
	Chicken	220	19 (8.6, 4.9–12.3)	0	-	0	-	-
	Stray dog	226	103 (45.6, 39.1–52.1)	0	-	0	-	-
	Stray cat	127	3 (2.4, 0.3–5.0)	0	-	0	-	-
	Donkey	40	33 (82.5, 70.7–94.3)	0	-	0	-	-
IgM	Yak	319	15 (4.7, 2.4–7.0)	257	27 (10.5, 6.8–14.3)	216	9 (4.2, 1.5–6.8)	0.0108
	Cow	489	13 (2.7, 1.2–4.1)	0	-	0	-	-
	Cattle	401	8 (2.0, 0.6–3.4)	0	-	50	1 (2.0, 1.9–5.9)	0.9981
	Tibetan sheep	147	23 (15.6, 9.8–21.5)	647	23 (3.6, 2.1–5.0)	0	-	<0.0001
	Horse	40	5 (12.5, 2.3–22.7)	289	22 (7.6, 4.6–10.7)	60	0	0.3390
	Pig	424	0	0	-	0	-	-
	Chicken	220	0	0	-	0	-	-
	Stray dog	226	68 (30.1, 24.1–36.1)	0	-	0	-	-
	Stray cat	127	3 (2.4, 0.3–5.0)	0	-	0	-	-

-, no tested; %: Prevalence; 95% CI: 95% Confidence Interval.

## Data Availability

All data generated or analyzed during this study are included in this published article.

## Supplementary Materials

The following supporting information can be downloaded at: https://www.mdpi.com/article/10.3390/ani12192723/s1, Table S1: Sampling sites in the study, Figure S1: The original results of the recombinant MSP5 protein of *Anaplasma* was expressed.

## References

[1] Anderson J.F., Magnarelli L.A. (2008). Biology of ticks. Infect. Dis. Clin. North. Am..

[2] Baneth G. (2014). Tick-borne infections of animals and humans: A common ground. Int. J. Parasitol..

[3] Lagunova E.K., Liapunova N.A., Tuul D., Otgonsuren G., Nomin D., Erdenebat N., Abmed D., Danchinova G.A., Sato K., Kawabata H. (2022). Co-infections with multiple pathogens in natural populations of *Ixodes persulcatus* ticks in Mongolia. Parasit. Vectors.

[4] Rar V., Tkachev S., Tikunova N. (2021). Genetic diversity of *Anaplasma* bacteria: Twenty years later. Infect. Genet. Evol..

[5] Battilani M., De Arcangeli S., Balboni A., Dondi F. (2017). Genetic diversity and molecular epidemiology of *Anaplasma*. Infect. Genet. Evol..

[6] Wang J., Kelly P., Zhang J., Shi Z., Song C., Zheng X., Zhang Y., Hao Y., Dong H., El-Mahallawy H.S. (2018). Detection of Dirofilaria immitis antigen and antibodies against *Anaplasma phagocytophilum*, *Borrelia burgdorferi* and *Ehrlichia canis* in dogs from ten provinces of China. Acta. Parasitol..

[7] Laamari A., Azzag N., Tennah S., Derdour S.Y., China B., Bouabdallah R., Ghalmi F. (2020). Seroprevalence of Antibodies Against *Anaplasma phagocytophilum* and *Borrelia burgdorferi* in Horses (*Equus caballus*) from Northern Algeria. J. Vet. Res..

[8] Selim A., Attia K.A., Alsubki R.A., Albohairy F., Kimiko I., Said M.B. (2022). The first study on the seroprevalence of *Anaplasma* spp. in small ruminants and assessment of associated risk factors in North Egypt. Vet. World.

[9] Yan Y., Lu C., Gong P., Pei Z., Peng Y., Jian F., Wang R., Zhang L., Qi M., Ning C. (2022). Molecular detection and phylogeny of *Anaplasma* spp. closely related to *Anaplasma phagocytophilum* in small ruminants from China. Ticks. Tick. Borne. Dis..

[10] He Y., Chen W., Ma P., Wei Y., Li R., Chen Z., Tian S., Qi T., Yang J., Sun Y. (2021). Molecular detection of *Anaplasma* spp., *Babesia* spp. and *Theileria* spp. in yaks (*Bos grunniens*) and Tibetan sheep (*Ovis aries*) on the Qinghai-Tibetan Plateau, China. Parasit. Vectors.

[11] Li J., Jian Y., Jia L., Galon E.M., Benedicto B., Wang G., Cai Q., Liu M., Li Y., Ji S. (2020). Molecular characterization of tick-borne bacteria and protozoans in yaks (*Bos grunniens*), Tibetan sheep (*Ovis aries*) and Bactrian camels (*Camelus bactrianus*) in the Qinghai-Tibetan Plateau Area, China. Ticks. Tick. Borne. Dis..

[12] Qi Y., Ai L., Zhu C., Lu Y., Lv R., Mao Y., Lu N., Tan W. (2022). Co-existence of Multiple *Anaplasma* Species and variants in ticks feeding on hedgehogs or cattle poses potential threats of anaplasmosis to humans and livestock in Eastern China. Front. Microbiol..

[13] Elhamiani Khatat S., Daminet S., Kachani M., Leutenegger C.M., Duchateau L., El Amri H., Hing M., Azrib R., Sahibi H. (2017). *Anaplasma* spp. in dogs and owners in north-western Morocco. Parasit. Vectors.

[14] Schäfer I., Kohn B. (2020). *Anaplasma phagocytophilum* infection in cats: A literature review to raise clinical awareness. J. Feline. Med. Surg..

[15] Saleem S., Ijaz M., Farooqi S.H., Ghaffar A., Ali A., Iqbal K., Mehmood K., Zhang H. (2018). Equine Granulocytic Anaplasmosis 28 years later. Microb. Pathog..

[16] Dzięgiel B., Adaszek Ł., Kalinowski M., Winiarczyk S. (2013). Equine granulocytic anaplasmosis. Res. Vet. Sci..

[17] Myczka A.W., Szewczyk T., Laskowski Z. (2021). The Occurrence of Zoonotic *Anaplasma phagocytophilum* Strains, in the Spleen and Liver of Wild Boars from North-West and Central Parts of Poland. Acta. Parasitol..

[18] Mason K.L., Gonzalez M.V., Chung C., Mousel M.R., White S.N., Taylor J.B., Scoles G.A. (2017). Validation of an improved *Anaplasma* antibody competitive ELISA for detection of *Anaplasma ovis* antibody in domestic sheep. J. Vet. Diagn. Investig..

[19] Bisen S., Aftab A., Jeeva K., Silamparasan M., Yadav S., Chandra D., Sankar M., Garg R., Raina O.K. (2021). Molecular and serological detection of Anaplasma infection in carrier cattle in north India. Vet. Parasitol. Reg. Stud. Rep..

[20] Noaman V., Sazmand A. (2021). *Anaplasma ovis* infection in sheep from Iran: Molecular prevalence, associated risk factors, and spatial clustering. Trop. Anim. Health Prod..

[21] Iqbal N., Mukhtar M.U., Yang J., Sajid M.S., Niu Q., Guan G., Liu Z., Yin H. (2019). First Molecular Evidence of *Anaplasma bovis* and *Anaplasma phagocytophilum* in Bovine from Central Punjab, Pakistan. Pathogens.

[22] Rajput Z.I., Hu S.H., Arijo A.G., Habib M., Khalid M. (2005). Comparative study of *Anaplasma* parasites in tick carrying buffaloes and cattle. J. Zhejiang Univ. Sci. B.

[23] Ismail N., McBride J.W. (2017). Tick-Borne Emerging Infections: Ehrlichiosis and Anaplasmosis. Clin. Lab. Med..

[24] Ndung’u L.W., Aguirre C., Rurangirwa F.R., McElwain T.F., McGuire T.C., Knowles D.P., Palmer G.H. (1995). Detection of *Anaplasma ovis* infection in goats by major surface protein 5 competitive inhibition enzyme-linked immunosorbent assay. J. Clin. Microbiol..

[25] Molloy J.B., Bowles P.M., Knowles D.P., McElwain T.F., Bock R.E., Kingston T.G., Blight G.W., Dalgliesh R.J. (1999). Comparison of a competitive inhibition ELISA and the card agglutination test for detection of antibodies to *Anaplasma marginale* and *Anaplasma centrale* in cattle. Aust. Vet J..

[26] Aubry P., Geale D.W. (2011). A review of bovine anaplasmosis. Transbound. Emerg. Dis..

[27] de Echaide S.T., Knowles D.P., McGuire T.C., Palmer G.H., Suarez C.E., McElwain T.F. (1998). Detection of cattle naturally infected with *Anaplasma marginale* in a region of endemicity by nested PCR and a competitive enzyme-linked immunosorbent assay using recombinant major surface protein 5. J. Clin. Microbiol..

[28] Dreher U.M., de la Fuente J., Hofmann-Lehmann R., Meli M.L., Pusterla N., Kocan K.M., Woldehiwet Z., Braun U., Regula G., Staerk K.D. (2005). Serologic cross-reactivity between *Anaplasma marginale* and *Anaplasma phagocytophilum*. Clin. Diagn. Lab. Immunol..

[29] Alleman A.R., Barbet A.F., Sorenson H.L., Strik N.I., Wamsley H.L., Wong S.J., Chandrashaker R., Gaschen F.P., Luckshander N., Bjöersdorff A. (2006). Cloning and expression of the gene encoding the major surface protein 5 (MSP5) of *Anaplasma phagocytophilum* and potential application for serodiagnosis. Vet. Clin. Pathol..

[30] Primo M.E., Thompson C.S., Valentini B.S., Sarli M., Novoa M.B., Mangold A.J., de Echaide S.T. (2019). Development of a novel fusion protein with *Anaplasma marginale* and A. centrale MSP5 improved performance of *Anaplasma* antibody detection by cELISA in infected and vaccinated cattle. PLoS ONE..

[31] Sarangi L.N., Rana S.K., Prasad A., Ponnanna N.M., Sharma G.K. (2021). Prevalence of antibodies to *Anaplasma* in cattle and buffaloes of different organized herds in India. J. Parasit. Dis..

[32] Rubel W., Schoneberg C., Wolf A., Ganter M., Bauer B.U. (2021). Seroprevalence and risk factors of *Anaplasma* spp. in German Small Ruminant Flocks. Animals.

[33] Sarli M., Thompson C.S., Novoa M.B., Valentini B.S., Mastropaolo M., Echaide I.E., de Echaide S.T., Primo M.E. (2020). Development and evaluation of a double-antigen sandwich ELISA to identify *Anaplasma marginale*-infected and *A. centrale*-vaccinated cattle. J. Vet. Diagn. Invest..

[34] Li H., Zheng Y.C., Ma L., Jia N., Jiang B.G., Jiang R.R., Huo Q.B., Wang Y.W., Liu H.B., Chu Y.L. (2015). Human infection with a novel tick-borne *Anaplasma* species in China: A surveillance study. Lancet. Infect. Dis..

[35] Han R., Yang J.F., Mukhtar M.U., Chen Z., Niu Q.L., Lin Y.Q., Liu G.Y., Luo J.X., Yin H., Liu Z.J. (2019). Molecular detection of *Anaplasma* infections in ixodid ticks from the Qinghai-Tibet Plateau. Infect. Dis. Poverty..

[36] de Echaide S.T., Bono M.F., Lugaresi C., Aguirre N., Mangold A., Moretta R., Farber M., Mondillo C. (2005). Detection of antibodies against *Anaplasma* marginale in milk using a recombinant MSP5 indirect ELISA. Vet. Microbiol..

[37] Cao W.C., Zhan L., He J., Foley J.E., DE Vlas S.J., Wu X.M., Yang H., Richardus J.H., Habbema J.D. (2006). Natural *Anaplasma phagocytophilum* infection of ticks and rodents from a forest area of Jilin Province, China. Am. J. Trop. Med. Hyg..

[38] Jiang J.F., Jiang B.G., Yu J.H., Zhang W.Y., Gao H.W., Zhan L., Sun Y., Zhang X.A., Zhang P.H., Liu W. (2011). *Anaplasma phagocytophilum* infection in ticks, China-Russia border. Emerg. Infect. Dis..

[39] Cazan C.D., Ionică A.M., Matei I.A., D’Amico G., Muñoz C., Berriatua E., Dumitrache M.O. (2020). Detection of *Leishmania infantum* DNA and antibodies against *Anaplasma* spp., *Borrelia burgdorferi* s.l. and *Ehrlichia canis* in a dog kennel in South-Central Romania. Acta. Vet. Scand..

[40] Zhang L., Liu H., Xu B., Lu Q., Li L., Chang L., Zhang X., Fan D., Li G., Jin Y. (2012). *Anaplasma phagocytophilum* infection in domestic animals in ten provinces/cities of China. Am. J. Trop. Med. Hyg..

[41] Xia Z., Yu D., Mao J., Zhang Z., Yu J. (2012). The occurrence of *Dirofilaria immitis*, *Borrelia burgdorferi*, *Ehrlichia canis* and *Anaplasma phagocytophilum* in dogs in China. J. Helminthol..

[42] Daniels T.J., Battaly G.R., Liveris D., Falco R.C., Schwartz I. (2002). Avian reservoirs of the agent of human granulocytic ehrlichiosis?. Emerg. Infect. Dis..

