# Supplementary material for: Serological Analysis of IgG and IgM Antibodies against Anaplasma spp. in Various Animal Species of the Qinghai-Tibetan Plateau
Source: Animals (Basel). 2022 Oct 10;12(19):2723. doi: 10.3390/ani12192723 (PMC9559258; doi:10.3390/ani12192723)
Supplement: Supplementary file 1 [file animals-12-02723-s001.zip › animals-1868999-Table S1.pdf]

Table S1. Sampling sites in the study

Prefectures	Sampling Sites	Latitude and longitude (N', E')	No. of Serum Samples *										
			Yak	Cow	Cattle	Tibetan Sheep	Pig	Chicken	Horse	Donkey	Stray Dog	Stray Cat	Total
Haibei	Menyuan (2866 m)	37.38, 101.61	20	0	11	36	0	0	0	0	0	0	67
	Gangcha (3827 m)	37.32, 100.14	0	0	0	404	0	0	14	0	0	0	418
	Haiyan (3000 m)	36.89, 100.99	104	0	0	45	0	0	10	0	0	0	159
	Total		124	0	11	485	0	0	24	0	0	0	644
Hainan	Gonghe (3200 m)	36.28, 100.62	20	0	0	190	0	0	265	0	0	0	475
	Guide (2200 m)	36.04, 101.43	0	0	0	0	30	0	0	0	0	0	30
	Total		20	0	0	190	30	0	265	0	0	0	505
Haixi	Delingha (2980 m)	37.36, 97.36	0	0	45	0	30	0	0	0	0	0	75
	Golmud (2780 m)	36.40, 94.90	20	0	0	0	20	0	0	0	0	0	40
	Tianjun (3993 m)	37.30, 99.02	0	0	0	8	0	0	0	0	0	0	8
	Total		20	0	45	8	50	0	0	0	0	0	123
Yushu	Zhiduo (4897 m)	33.84, 95.61	20	0	0	0	0	0	0	0	0	0	20
	Total		20	0	0	0	0	0	0	0	0	0	20
Guoluo	Maqin (4100 m)	34.47, 100.23	110	0	50	0	0	0	0	0	0	0	160
	Darlag (4271 m)	33.74, 99.65	86	0	0	0	0	0	0	0	0	0	86
	Banma (3970 m)	32.93, 100.73	133	0	0	0	0	0	0	0	0	0	133
	Total		329	0	50	0	0	0	0	0	0	0	379
Huangnan	Jianzha (2063 m)	35.93, 102.03	0	0	45	45	0	0	0	0	0	0	90
	Henan (4000 m)	34.73, 101.61	0	0	0	0	0	0	60	0	0	0	60
	Total		0	0	45	45	0	0	60	0	0	0	150
Haidong	Huzhu (2535 m)	36.84, 101.95	40	0	0	21	48	30	0	40	0	0	179
	Ledu (2000 m)	36.48, 102.40	20	282	100	0	67	0	0	0	0	0	469
	Minhe (2174 m)	36.32, 102.83	0	207	0	0	0	30	0	0	0	0	237
	Pingan (2183 m)	36.50, 102.10	0	0	0	45	77	0	0	0	0	0	122
	Total		60	489	100	66	192	60	0	40	0	0	1007
Xining	Datong (2756 m)	36.92, 101.68	219	0	200	0	30	80	30	0	0	0	559
	Huangzhong (2645 m)	36.50, 101.57	0	0	0	0	30	50	10	0	0	0	90
	Huangyuan (2660 m)	36.68, 101.25	0	0	0	0	48	30	0	0	0	0	78
	Xining (2261 m)	36.65, 101.76	0	0	0	0	44	0	0	0	226	127	397
	Total		219	0	200	0	152	160	40	0	226	127	1124
Total			792	489	451	794	424	220	389	40	226	127	3952
