# Supplementary figures and images for: Serological Analysis of IgG and IgM Antibodies against Anaplasma spp. in Various Animal Species of the Qinghai-Tibetan Plateau
Source: Animals (Basel). 2022 Oct 10;12(19):2723. doi: 10.3390/ani12192723 (PMC9559258; doi:10.3390/ani12192723)

Figure S1

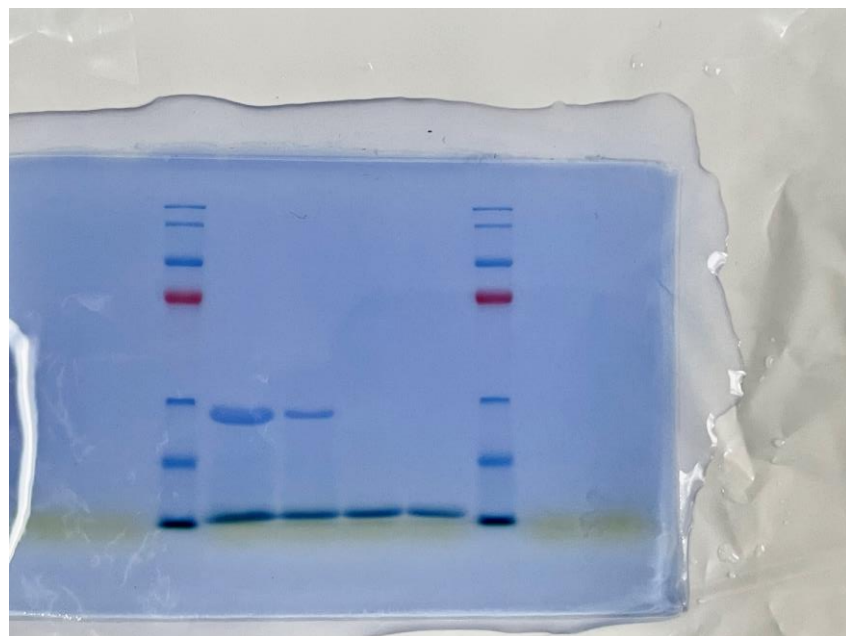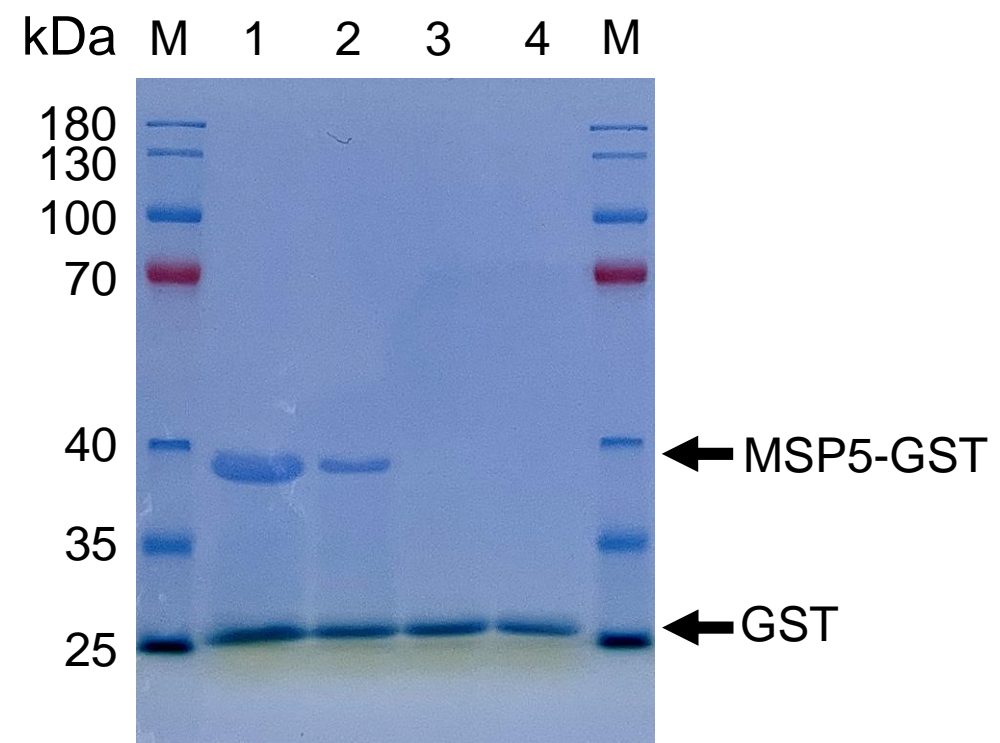

Supplement: Supplementary file 1 [file animals-12-02723-s001.zip › animals-1868999-Figure S1.pdf]
